# Heart rate variability status at rest in adult depressed patients: a systematic review and meta-analysis

**DOI:** 10.3389/fpubh.2023.1243213

**Published:** 2023-12-19

**Authors:** Qianqian Wu, Xiangyang Miao, Yingying Cao, Aiping Chi, Tao Xiao

**Affiliations:** ^1^School of Physical Education, Shaanxi Normal University, Xi’an, China; ^2^Southwest University, Chongqing, China

**Keywords:** depression, heart rate variability, meta-analysis, case-control study, adult

## Abstract

**Purposes:**

A meta-analysis was conducted to examine the differences in heart rate variability (HRV) between depressed patients and healthy individuals, with the purpose of providing a theoretical basis for the diagnosis of depression and the prevention of cardiovascular diseases.

**Methods:**

To search China National Knowledge Infrastructure (CNKI), WanFang, VIP, PubMed, Web of Science, Science Direct, and Cochrane Library databases to collect case–control studies on HRV in depressed patients, the retrieval date is from the establishment of the database to December 2022. Effective Public Health Practice Project (EPHPP) scale was used to evaluate literature quality, and Stata14.0 software was used for meta-analysis.

**Results:**

This study comprised of 43 papers, 22 written in Chinese and 21 in English, that included 2,359 subjects in the depression group and 3,547 in the healthy control group. Meta-analysis results showed that compared with the healthy control group, patients with depression had lower SDNN [*Hedges’ g* = −0.87, *95% CI* (−1.14, −0.60), *Z* = −6.254, *p* < 0.01], RMSSD [*Hedges’ g* = −0.51, *95% CI* (−0.69,-0.33), *Z* = −5.525, *p* < 0.01], PNN50 [*Hedges’ g* = −0.43, *95% CI* (−0.59, −0.27), *Z* = −5.245, *p* < 0.01], LF [*Hedges’ g* = −0.34, *95% CI* (−0.55, − 0.13), *Z* = −3.104, *p* < 0.01], and HF [*Hedges’ g* = −0.51, *95% CI* (−0.69, −0.33), *Z* = −5.669 *p* < 0.01], and LF/HF [*Hedges’ g* = −0.05, *95% CI* (−0.27, 0.18), *Z* = −0.410, *p* = 0.682] showed no significant difference.

**Conclusion:**

This research revealed that HRV measures of depressed individuals were lower than those of the healthy population, except for LF/HF, suggesting that people with depression may be more at risk of cardiovascular diseases than the healthy population.

## 1 Introduction

Depression is a mental health condition characterized by prolonged feelings of sadness and lack of interest in activities. People suffering from this disorder may have decreased appetite, be unresponsive, experience physical discomfort and even thoughts of suicide (1). With the world advancing, the rate of depression has been on the rise, with 11.7% of people in China estimated to be affected (2). By the year 2020, depression had become the second most widespread medical condition globally (3). Besides causing certain shared symptomatic responses, depression may also affect the autonomic performance of the heart (4).

Heart rate variability (HRV), which refers to the magnitude of the difference in fluctuations between adjacent R-R intervals, is a noninvasive monitoring tool that can be used to assess the balance of sympathetic and vagal tone and autonomic activity in the heart (5). The combination of the sympathetic and parasympathetic nervous systems is essential for the maintenance of the autonomic balance of the heart, and if this balance is disrupted, HRV can be used to measure the degree of pathology (6). The SDNN index is deemed the “gold standard” for predicting the gravity of cardiac diseases and mortality rates (7), and it is feasible to evaluate an individual’s well-being through prolonged testing of the SDNN index (8); RMSSD is indicative of alterations mediated by the vagus nerve; PNN50 is strongly associated with vagal nerve activity; The frequency domain index LF is thought to mirror the equilibrium between sympathetic and vagal nerves; HF is affected by respiratory regulation and may be indicative of parasympathetic activity and vagal function; Whether LF/HF is a reliable indicator of the balance between sympathetic and vagal nerves is still a matter of debate. Depression is a potential risk factor of cardiovascular disease, and the autonomous nervous system is believed to be connected to a wide range of physical and mental conditions.

Research has indicated that there is a significant link between HRV and cardiovascular disorders, such as coronary heart disease and heart failure. Furthermore, a decrease in HRV is usually a sign of damage to the autonomic nervous system (9). Compared to healthy individuals, those suffering from depression have a greater risk of developing cardiovascular diseases, and those with CVD are more likely to experience depression than those without, forming a two-way relationship between depression and CVD (10). Due to the fact that HRV contains a wealth of data concerning cardiovascular regulation, it can be utilized to effectively monitor the cardiovascular functioning of individuals suffering from depression. In this study, we conducted a meta-analysis of published controlled experiments on HRV in depressed patients and healthy subjects in order to gain a better understanding of the changes in HRV in depressed patients via a systematic analysis, which is anticipated to provide an objective foundation for the diagnosis and treatment of depressed patients and the prevention of cardiovascular diseases.

## 2 Materials and methods

### 2.1 Search strategy

This systematic review was conducted according to the Preferred Reporting of Systematic Reviews and Meta Analyses (PRISMA) guidelines (11). In this paper, two researchers searched PubMed, Science Direct, Web Of Science, Cochrane, CNKI, WanFang, and VIP databases by computer, using a mixed search of subject terms and free words in English and Chinese, and the search terms were mainly (Depression or Depression or Depressive Symptoms or Depressive Symptom or Symptom, Depressive or Emotional Depression or Depression, Emotional) and (Heart Rate Variability or HRV or autonomic nervous system or cycle length variability or RR variability or heart period variability or vagal or ANS). To make the search more comprehensive, we also performed a manual search from published meta-analyses to literature reviews. The search was conducted from the date of database creation to December, 2022. Take PubMed as an example, its specific search strategy was ((“Depression”[Mesh]) OR (((((Depressive Symptoms [Title/Abstract]) OR (Depressive Symptom [Title/Abstract])) OR (Symptom, Depressive [Title/Abstract])) OR (Emotional Depression [Title/Abstract])) OR (Depression, Emotional [Title/Abstract]))) AND (((((((Heart Rate Variability [Title/Abstract]) OR (HRV [Title/Abstract])) OR (autonomic nervous system [Title/Abstract] OR cycle length variability [Title/Abstract])) OR (RR variability [Title/Abstract])) OR (heart period variability [Title/Abstract])) OR (vagal [Title/Abstract])) OR (ANS [Title/Abstract])).

### 2.2 Literature screening process

Two pre-trained researchers independently conducted a literature screening process, adhering to the inclusion and exclusion criteria, and then combined the chosen literature. In the event of conflicting conclusions between the two researchers, the discrepancies were initially addressed through dialog, and if the disagreement persisted, a third-party specialist was consulted to make a final decision, ensuring agreement was reached prior to inclusion. According to PICOS, that is, the study population, intervention, comparison, outcome and study design were used for literature screening.

Inclusion criteria: (1) published journals, conference papers, and dissertations at home and abroad; (2) the experimental group was patients who met the assessment criteria of the depression scale or had been clinically diagnosed with depression, and the control group was the healthy general population; (3) the study type was a controlled trial; (4) the outcome indicators were SDNN, RMSSD, and PNN50 in the time domain, and LF, HF, and LF/HF in the frequency domain.

Exclusion criteria: (1) Exclusion of review articles, animal experiment articles, and articles where full text and outcome indicators were not available; (2) Exclusion of articles with depression co-morbidities such as diabetes, hypertension, heart disease, and other serious physical diseases; (3) Excluded patients who had taken antidepressants or medications that affected heart rate in the recent past.

### 2.3 Data extraction

Researchers conducted a thorough search of each database, applied the inclusion and exclusion criteria to the literature, and then summarized the findings. In the event of any discrepancies, these were discussed and resolved. If the problem still could not be solved, the third-party experts would make a ruling, and the inclusion could only be reached by consensus. The content of literature data extraction mainly includes: (1) general characteristics of literature (first author, publication year, country); (2) Characteristics of subjects (gender, age, sample size, diagnostic scale, etc.); (3) The mean and standard deviation of each outcome index.

### 2.4 Quality assessment

This research has made Effective Public Health Practice Project (EPHPP) scale was biased risk assessment (12). The scale included evaluations of selection bias, study design, confounding factors, blinding, data collection, and inclusion and exclusion, each rated as weak, medium, and strong. According to the number of sub-items rated as weak, the overall evaluation is divided into three levels: strong (no weak items), medium (1 weak item), and weak (2 or more weak items). The retrieval, inclusion and quality evaluation of the literature are carried out by two authors, who discuss the results of the operation and make a decision. If no consensus can be reached through discussion, the results will be submitted to the third author for consultation before making a decision.

### 2.5 Statistical methods

Data were statistically analyzed using Stata14.0 software, and effect sizes were calculated using mean and standard variance (Mean ± SD). The effect size employed was Hedges’ g, which is a modification of Cohen’s d to account for a small sample size. A Hedges’ g value of 0.2 ~ 0.5 indicates a low effect, 0.5 ~ 0.8 indicates moderate effect, and greater than 0.8 indicates significant effect. Although the people included in this study were patients with depression, there were many types of depression, which were not subdivided in this study, so we chose the random effects model. *Q* test was used to examine the heterogeneity among the included references. In *Q* test, when *p* < 0.01, the heterogeneity was considered significant (13). However, since *Q* value cannot judge the degree of heterogeneity among studies, *I^2^* is further used to describe the proportion of variance between studies in the total variance. According to Cochrane systematic reviews, heterogeneity among studies can be ignored when *I^2^* is less than 50%. If *I^2^* is greater than 50%, heterogeneity between studies cannot be ignored (14). Subgroup analysis was used to explore the source of heterogeneity. Sensitivity analysis was carried out to check the stability of the results by using the one-by-one elimination method and model transformation. Publication bias was evaluated by Egger test and funnel plot. If there was publication bias, the effect value was corrected by non-parametric trim and fill procedure.

## 3 Results

### 3.1 Results of the literature search

**Figure 1:**
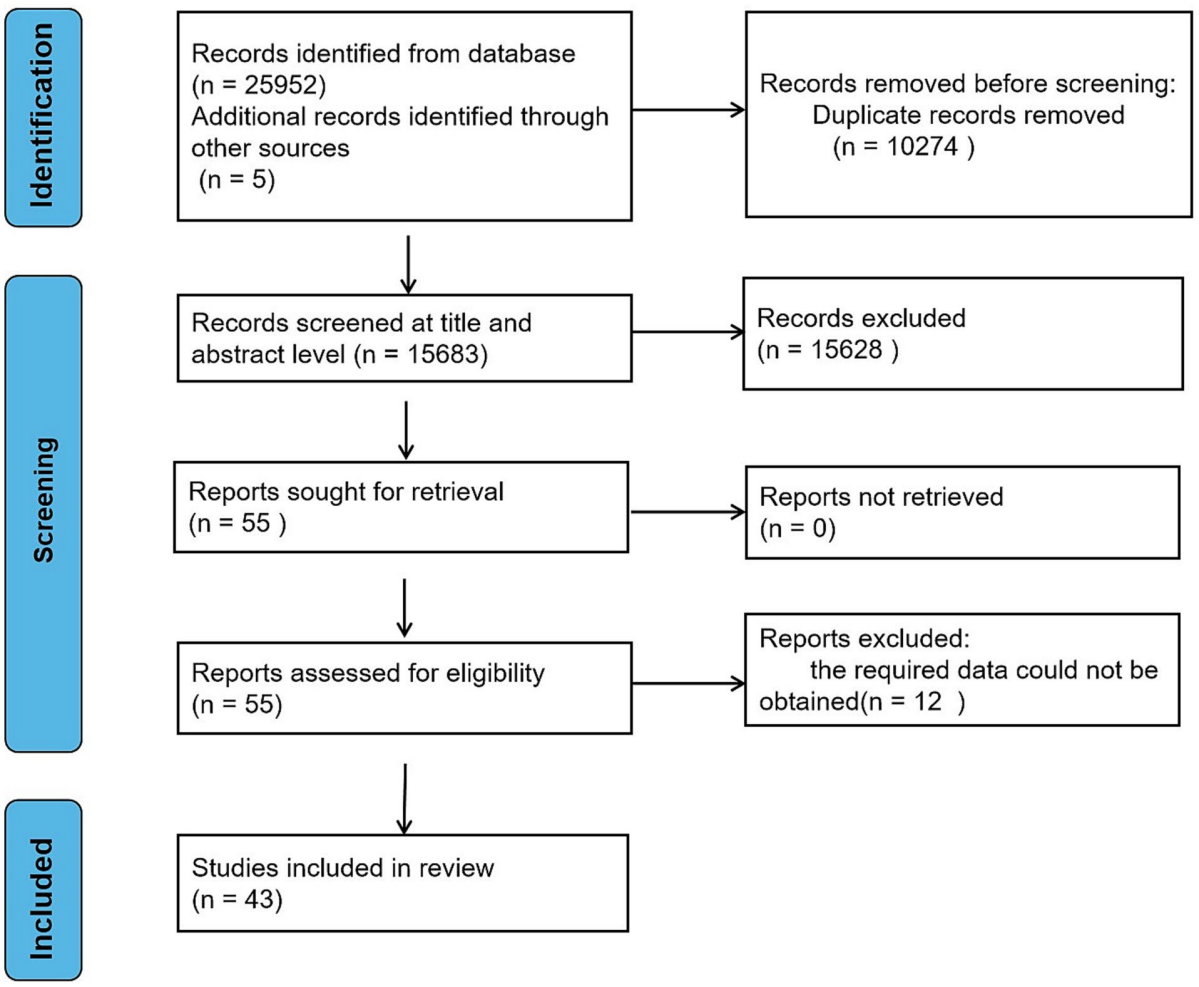
Flow chart of literature search.

A total of 25,957 articles were retrieved through PubMed, Science Direct, Web Of Science, Cochrane, CNKI, WanFang, and VIP databases. Following the removal of duplicate published literature, 15,683 articles were obtained; upon preliminary reading of titles and abstracts, 1,295 articles were retained after the exclusion of reviews, policy research, results category, less relevant research and inaccessible articles. Moreover, through reading the full text to discard any unqualified literature, 43 references were included. The literature screening process is illustrated in Figure 1.

### 3.2 Research characteristics

The basic information of all included literature was shown in Table 1, a total of 43 papers were included, 22 (15–36) in Chinese and 21 (37–54) in English. However, 24 (15–36, 42, 51) of the studies involved Chinese people, and 19 (37–41, 43–50, 52–54) involved participants from countries other than China. Among them, 2,359 were in the depression group and 3,547 were in the healthy control group, and the years of publication were between 2000 and 2021. 26 (15–20, 22–37, 42, 44, 45, 47, 55) studies reported the outcome indicator SDNN. 28 (15–26, 28, 29, 31, 33–35, 38, 40–42, 44, 45, 47, 49, 50, 52, 55) studies reported the outcome indicator RMSSD. 13 (14, 15, 22, 25–27, 29, 31, 33–35, 42, 48, 50) studies reported the outcome indicator PNN50. The outcome indicator LF was reported in 27 (15, 17–23, 27, 29–32, 34, 35, 38, 39, 42, 43, 45, 48, 50, 51, 54, 56) studies. 31 (15, 17–23, 27, 29–32, 34, 38–43, 45–51, 54–56) studies reported the outcome indicator HF. 31 (15, 17–19, 21–24, 28–32, 34–36, 38–46, 48–57) studies reported the outcome indicator LF/HF. The overall quality of two (26, 33) of the articles was weak, and the overall quality of the others was moderate (15–27, 29–34, 36–57).

**Table 1:** Basic information of the included literature.

Author and year	Country	Diagnostic criteria	Depression group gender/Number of people/Age	Healthy control group gender/Number of people/Age	Ending indicators
Guan et al. (2021) (15)	China	DSM-V/SDS	♂38♀49/87/23.59 ± 3.90	♂47♀37/84/22.74 ± 2.28	a, b, c, d, e, f
Li et al. (2021) (16)	China	CCMD-3	♂9♀29/38/46.6 ± 16.8	♂14♀18/32/40.4 ± 14.6	a, b
Wang (2021) (17)	China	HAMD	♂63♀83/146/44.47 ± 14.61	♂15♀24/39/45.36 ± 14.83	a, b, c, d, e, f
Chen (2021) (18)	China	HAMD	♂42♀53/95/73.6 ± 7.88	♂29♀31/60/74.10 ± 8.10	a, b, d, e, f
Wu et al. (2020) (19)	China	ICD-10	♂37♀21/48/39.21 ± 10.08	♂27♀22/49/39.24 ± 10.90	a, b, d, e, f
Zhao (2020) (20)	China	BDI/SDS	♀/12/18.46 ± 0.51	♀/12/18.82 ± 0.36	a, b, d, e
Liu (2020) (21)	China	CCMD-3/HAMD	♂8♀19/27/41.11 ± 13.24	♂9♀15/24/44.25 ± 12.57	d, e, f
Kuang (2019) (22)	China	HAMD	♂58♀33/91/29.8 ± 10.0	♂58♀33/91/26.3 ± 4.2	a, b, c, d, e, f
Liu et al. (2019) (23)	China	ICD-10/HAMD	♂11♀19/30/45.8 ± 4.0	♂18♀32/50/46.5 ± 4.8	a, b, d, e, f
Fu et al. (2019) (24)	China	EPDS	♀/47/ 42.78 ± 5.44	♀243/ 42.53 ± 5.71	a, b, f
Sun (2019) (25)	China	Patients with confirmed depression	♂46♀54/100/57.09 ± 16.29	♂48♀56/104/53.31 ± 10.18	a, b, c
Zhang et al. (2018) (26)	China	Patients with confirmed depression	♀/35/26.58 ± 4.87	♀/30/25.88 ± 4.35	a, b, c
Yang (2018) (27)	China	ICD-10	♂11♀32/43/31.2 ± 10.4	♂11♀32/43/32.5 ± 11.4	b, c, d, e
Zhao and Zhao (2017) (28)	China	EPDS	♀/39/>20	♀/128/>20	a, b, f
Li et al. (2017) (29)	China	SCL-90/ICD-10/HAMD	♂39♀69/108	119	a, b, c, d, e, f
Wu et al. (2015) (30)	China	CCMD-3/SCL-90/Zung	♂15♀16/31/67.2 ± 5.6	♂14♀14/28/66.8 ± 5.3	a, d, e, f
Shao et al. (2014) (31)	China	ICD-10/HAMD	♂14♀20/34/36 ± 12	♂14♀21/35/37 ± 10	a, b, c, d, e, f
Xuan (2014) (32)	China	CCMD-3/HAMD	♂18♀21/39/39.32 ± 16.2	♂20♀21/41/42.64 ± 15.21	a, d, e, f
Wang (2013) (33)	China	CCMD-3	♂36♀44/80/58.01 ± 17.03	♂38♀42/80/54.32 ± 11.09	a, b, c
Zhang et al. (2008) (34)	China	CCMD-3	22/45.32 ± 15.46	♂109♀138/247/37.89 ± 14.16	a, b, c, d, e, f
Feng and Feng (2007) (35)	China	CCMD-3	♂10女14/24/42 ~ 70	♂12♀11/23/45 ~ 65	a, b, d, f
Li and Li (2007) (36)	China	CCMD-3/SDS	♂13♀18/31/36 ± 10.49	♂12♀19/31/36 ± 11.60	f
Smith et al. (2020) (37)	United Kingdom	Patients with confirmed depression	♂4♀7/11/43.5 ± 9.8	♂5♀11/16/41.4 ± 12.4	a
Lim et al. (2020) (38)	Korea	PHQ-9	♀/19/ 24.3 ± 4.0	♀/27/ 24.2 ± 3.8	b, d, e, f
Tsujita et al. (2020) (39)	Japan	CES-D	♂14♀21/35/20.5 ± 2.1	35/20.3 ± 1.6	d, e, f
Baik et al. (2019) (40)	Korea	DSM-V	38/42.24 ± 13.76	34/37.37 ± 13.28	b, e, f
Caldwell and Steffen (2018) (41)	United States	MINI/BDI	♀/10/18–25	♀/10/18–25	b, e, f
Chen et al. (2017) (42)	China	DSM-IV	♂25♀15/40/35.0 ± 12.4	♂25♀15/40/36.1 ± 9.6	a, b, c, d, e, f
Shinba (2017) (43)	Japan	DSM-IV/SDS	♂7♀7/14/38.5 ± 10.0	♂18♀23/41/39.4 ± 11.9	d, e, f
Schumann et al. (2017) (44)	Germany	BDI/HAMD	♂8♀21/29/37.8 ± 12.2	♂8♀21/29/36.9 ± 12.5	a, b, f
Khandoker et al. (2016) (45)	United Arab Emirates	MINI/HAMD/BDI	♂3♀13/32.31 ± 6.95	♂12♀17/28.00 ± 6.35	a, b, d, e, f
Shinba (2014) (46)	Japan	DSM-IV/SDS	♂10♀12/22/39.5 ± 10.6	♂21♀26/47/40.7 ± 12.2	e, f
Brunoni et al. (2013) (47)	Brazil	MINI	♂38♀80/118/42.27 ± 12.6	♂38♀82/120/42.9 ± 13.17	b, e
Dauphinot et al. (2012) (48)	France	QD2A	♂8♀44/52/ 65.62 ± 8.9	♂324♀388/712/65.65 ± 8.0	a, c, d, e
Berger et al. (2012) (49)	Germany	DSM-IV/ HAMD/BDI	♂6♀12/18/ 43.83 ± 14.02	♂7♀11/18/42.22 ± 14.62	b, e, f
Garcia et al. (2012) (50)	Colombia	Zung /DSM-IV	♀34/22.3 ± 5.1	♀34/23.5 ± 5.7	b, c, d, e, f
Chang et al. (2011) (51)	China	DSM-IV/ HAMD	♂249♀249/498/39.13 ± 14.12	♂238♀224/462/40.66 ± 14.89	d, e, f
Berger et al. (2011) (52)	Germany	DSM-IV/ HAMD	♂12♀18/30/34.37 ± 12.83	♂12♀18/30/ 33.13 ± 10.60	b, f
Voss et al. (2011) (53)	Germany	DSM-IV/ HAMD/BDI	♂18♀18/36/25–41	♂18♀18/36/25–43	f
Kikuchi et al. (2009) (54)	Japan	DSM-IV/ HAMD	♂9♀6/15/20–50	♂8♀7/15/21–59	d, e, f
Udupa et al. (2007) (55)	India	DSM-IV/ HAMD	♂26♀14/40/30.58 ± 7.4	♂26♀14/40/30.73 ± 7.1	a, b, e, f
Yeragani et al. (2002) (56)	India	DSM-III-R/ HAMD	♂3♀11/14/35.4 ± 6.2	♂8♀10/18/34.7 ± 7.1	d, e, f
Yeragani et al. (2000) (57)	India	DSM-III-R	♂11♀25/36/31.7 ± 5.9	♂16♀18/30.2 ± 7.1	f

♂ = male; ♀ = female; CCMD-3, Chinese Classification and Diagnostic Standard of Mental Disorders, 3rd Edition; HAMD, Hamilton Depression Scale; SDS, Self-Rating Depression Scale; ICD-10, International Classification of Diseases; SCL-90, Symptom Checklist90; Zung, Zung’s Self-Rating Depression Scale; DSM-V, Diagnostic and Statistical Manual of Mental Disorders, 5th Edition; BDI, Beck Depression Inventory; EPDS, Edinburgh Postnatal Depression Scale; MINI, Mini International Neuropsychiatric Interview; PHQ-9, Patient Health Questionnaire-9; DSM-IV, Diagnostic and Statistical Manual of Mental Disorders, 4rd Edition; DSM-III-R, Psychiatry Diagnostic & Statistical Manual of Mental Disorders 3rd Edition; CES-D, Center for Epidemiological Survey, Depression Scale; a, SDNN (standard deviation of R-R intervals); b, RMSSD (root mean square of the difference between adjacent R-R intervals); c, PNN50 (RR50 divided by the total number of RR intervals multiplied by 100); d, LF (low frequency power); e, HF (high frequency power); f, LF/HF (low frequency power/high frequency power).

### 3.3 Risk of bias estimation

The results of the risk of bias assessment are shown in Table 2.

**Table 2:** Quality assessment of included studies according to the EPHPP.

Included literature	Selection bias	Research design	Confounding factor	Blind method	Data collection method	Loss of follow-up and withdrawal	Overall quality
Guan et al. (2021) (15)	Strong	Strong	Strong	Weak	Strong	Strong	Medium
Li et al. (2021) (16)	Strong	Strong	Medium	Weak	Strong	Strong	Medium
Wang (2021) (17)	Strong	Strong	Medium	Weak	Strong	Strong	Medium
Chen (2021) (18)	Strong	Strong	Strong	Weak	Strong	Strong	Medium
Wu et al. (2020) (19)	Strong	Strong	Strong	Weak	Strong	Strong	Medium
Zhao (2020) (20)	Strong	Strong	Medium	Weak	Strong	Strong	Medium
Liu (2020) (21)	Strong	Strong	Strong	Weak	Strong	Strong	Medium
Kuang (2019) (22)	Strong	Strong	Medium	Weak	Strong	Strong	Medium
Liu et al. (2019) (23)	Strong	Strong	Medium	Weak	Strong	Strong	Medium
Fu et al. (2019) (24)	Strong	Strong	Strong	Weak	Strong	Strong	Medium
Sun (2019) (25)	Strong	Strong	Strong	Weak	Strong	Strong	Medium
Zhang et al. (2018) (26)	Strong	Strong	Strong	Weak	Strong	Strong	Medium
Yang (2018) (27)	Strong	Strong	Strong	Weak	Strong	Strong	Medium
Zhao and Zhao (2017) (28)	Strong	Strong	Weak	Weak	Strong	Strong	Weak
Li et al. (2017) (29)	Strong	Strong	Strong	Weak	Strong	Strong	Medium
Wu et al. (2015) (30)	Strong	Strong	Strong	Weak	Strong	Strong	Medium
Shao et al. (2014) (31)	Strong	Strong	Strong	Weak	Strong	Strong	Medium
Xuan (2014) (32)	Strong	Strong	Medium	Weak	Strong	Strong	Medium
Wang (2013) (33)	Strong	Strong	Strong	Weak	Strong	Strong	Medium
Zhang et al. (2008) (34)	Strong	Strong	Strong	Weak	Strong	Strong	Medium
Feng and Feng (2007) (35)	Strong	Strong	Weak	Weak	Strong	Strong	Weak
Li and Li (2007) (36)	Strong	Strong	Medium	Weak	Strong	Strong	Medium
Smith et al. (2020) (37)	Strong	Strong	Strong	Weak	Strong	Strong	Medium
Lim et al. (2020) (38)	Strong	Strong	Strong	Weak	Strong	Strong	Medium
Tsujita et al. (2020) (39)	Strong	Strong	Strong	Weak	Strong	Strong	Medium
Baik et al. (2019) (40)	Strong	Strong	Strong	Weak	Strong	Strong	Medium
Caldwell and Steffen (2018) (41)	Strong	Strong	Medium	Weak	Strong	Strong	Medium
Chen et al. (2017) (42)	Strong	Strong	Medium	Weak	Strong	Strong	Medium
Shinba (2017) (43)	Strong	Strong	Strong	Weak	Strong	Strong	Medium
Schumann et al. (2017) (44)	Strong	Strong	Strong	Weak	Strong	Strong	Medium
Khandoker et al. (2016) (45)	Strong	Strong	Strong	Weak	Strong	Strong	Medium
Shinba (2014) (46)	Strong	Strong	Strong	Weak	Strong	Strong	Medium
Brunoni et al. (2013) (47)	Strong	Strong	Strong	Weak	Strong	Strong	Medium
Dauphinot et al. (2012) (48)	Strong	Strong	Strong	Weak	Strong	Strong	Medium
Berger et al. (2012) (49)	Strong	Strong	Strong	Weak	Strong	Strong	Medium
Garcia et al. (2012) (50)	Strong	Strong	Strong	Weak	Strong	Strong	Medium
Chang et al. (2011) (51)	Strong	Strong	Strong	Weak	Strong	Strong	Medium
Berger et al. (2011) (52)	Strong	Strong	Strong	Weak	Strong	Strong	Medium
Voss et al. (2011) (53)	Strong	Strong	Strong	Weak	Strong	Strong	Medium
Kikuchi et al. (2009) (54)	Strong	Strong	Strong	Weak	Strong	Strong	Medium
Udupa et al. (2007) (55)	Strong	Strong	Strong	Weak	Strong	Strong	Medium
Yeragani et al. (2002) (56)	Strong	Strong	Strong	Weak	Strong	Strong	Medium
Yeragani et al. (2000) (57)	Strong	Strong	Strong	Weak	Strong	Strong	Medium

EPHPP, Effective Public Health Practice Project.

### 3.4 Results of meta-analysis

#### 3.4.1 SDNN results

**Figure 2:**
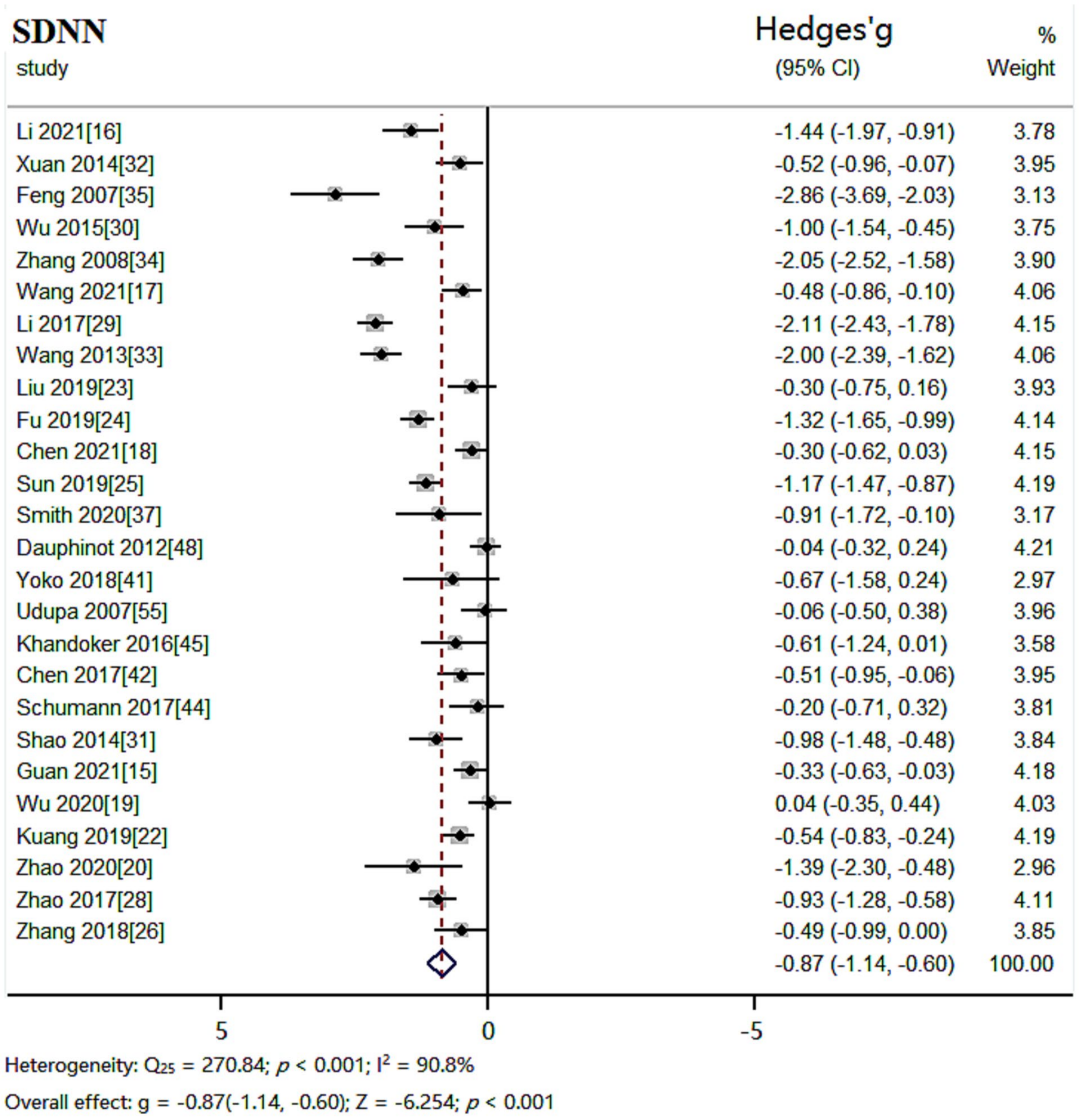
Meta-analysis forest plot of indicator SDNN.

The random effects model was used for meta-analysis of outcome index SDNN. Heterogeneity test results showed: *Q_25_* = 270.84, *p* < 0.001, *I^2^* = 90.8%, the heterogeneity between the studies was significant. Combined effect size: *Hedges’ g* = −0.87, *95% CI* (−1.14, −0.60), *Z* = −6.254, *p* < 0.001. The effect size was large. The forest plot (Figure 2) showed that the horizontal line of the 95% CI for the indicator Hedges’ g was to the left of the null line for the depressed and healthy control groups, indicating that the SDNN was significantly lower in the depressed group compared to the healthy control group.

#### 3.4.2 RMSSD results

**Figure 3:**
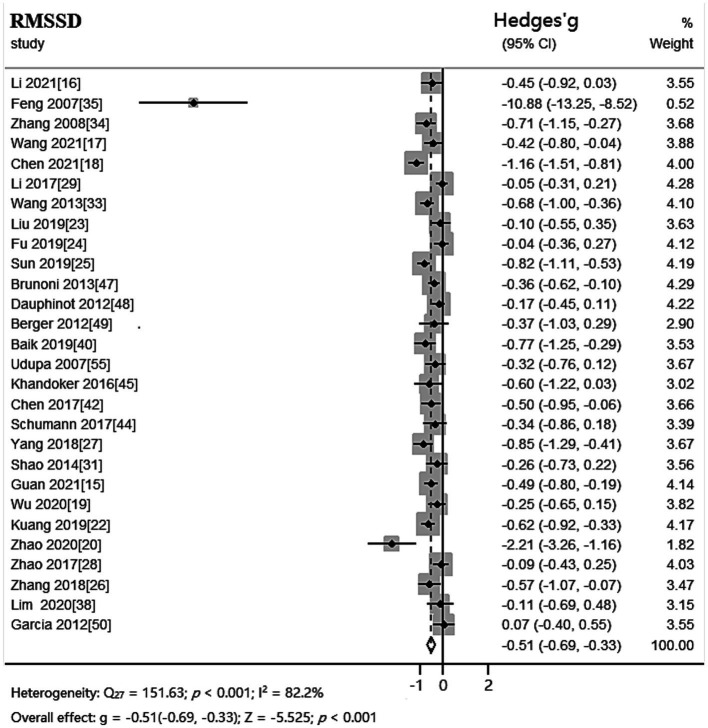
Meta-analysis forest plot of indicator RMSSD.

The random effects model was used for meta-analysis of outcome index RMSSD. Heterogeneity test results showed: *Q_27_* = 151.63, *p* < 0.001, *I^2^* = 82.2%, the heterogeneity between the studies was significant. Combined effect size: *Hedges’ g* = −0.51, *95% CI* (−0.69, −0.33), *Z* = −5.525, *p* < 0.001. The effect size was moderate. The forest plot showed (Figure 3) that the horizontal line of the 95% CI for the indicator Hedges’ g was to the left of the null line in the depressed and healthy control groups, indicating a significantly lower RMSSD in the depressed group compared to the healthy control group.

#### 3.4.3 PNN50 results

**Figure 4:**
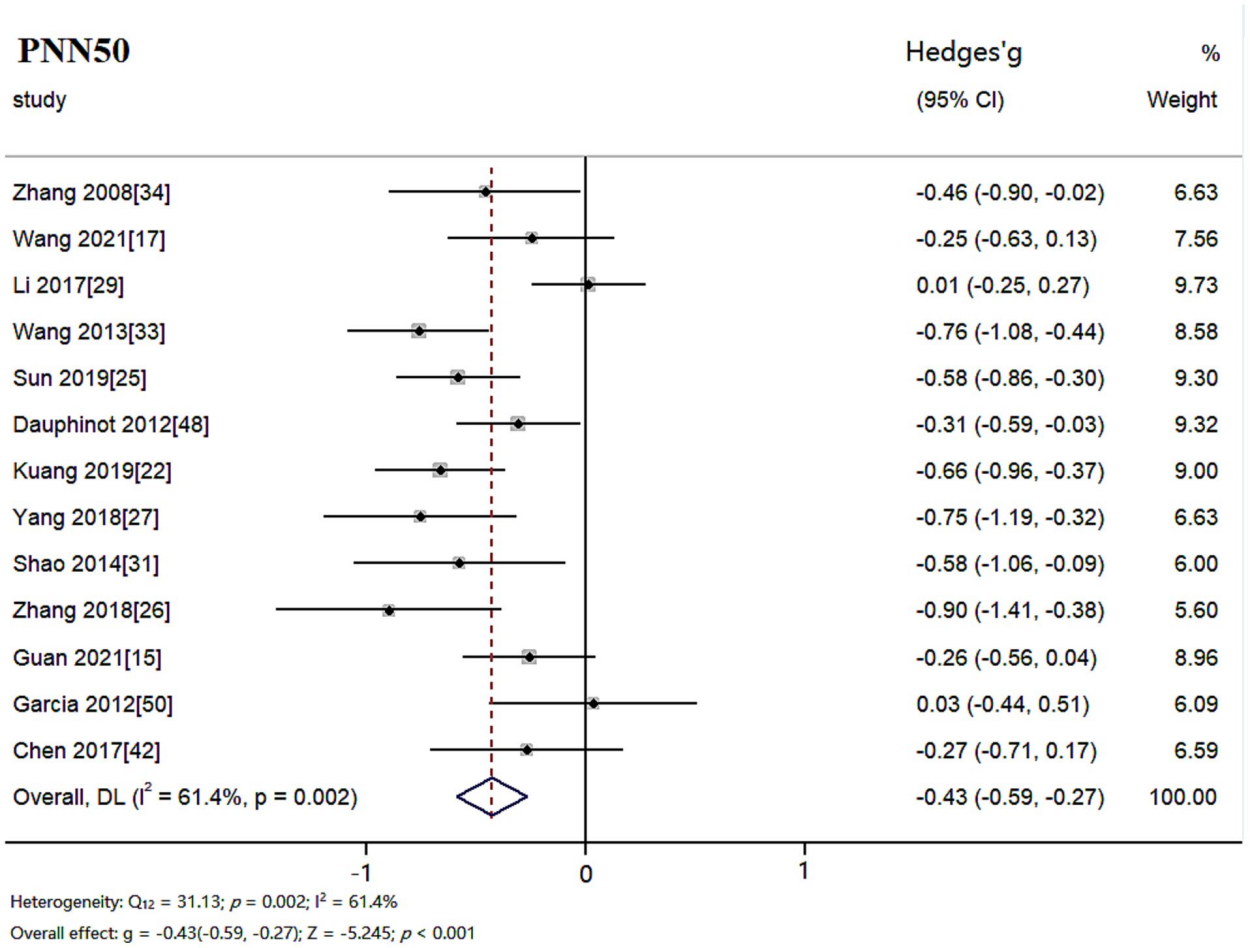
Meta-analysis forest plot of indicator PNN50.

The random effects model was used for meta-analysis of outcome index PNN50. Heterogeneity test results showed: *Q_12_* = 31.13, *p* = 0.002, *I^2^* = 61.4%, the heterogeneity between the studies was significant. Combined effect size: *Hedges’ g* = −0.43, *95% CI* (−0.59, −0.27), *Z* = −5.245, *p* < 0.001. The effect size was small. The forest plot showed (Figure 4) that the horizontal line of the 95% CI for the indicator Hedges’ g was to the left of the null line for the depressed and healthy control groups, indicating that the PNN50 was significantly lower in the depressed group compared to the healthy control group.

#### 3.4.4 LF results

**Figure 5:**
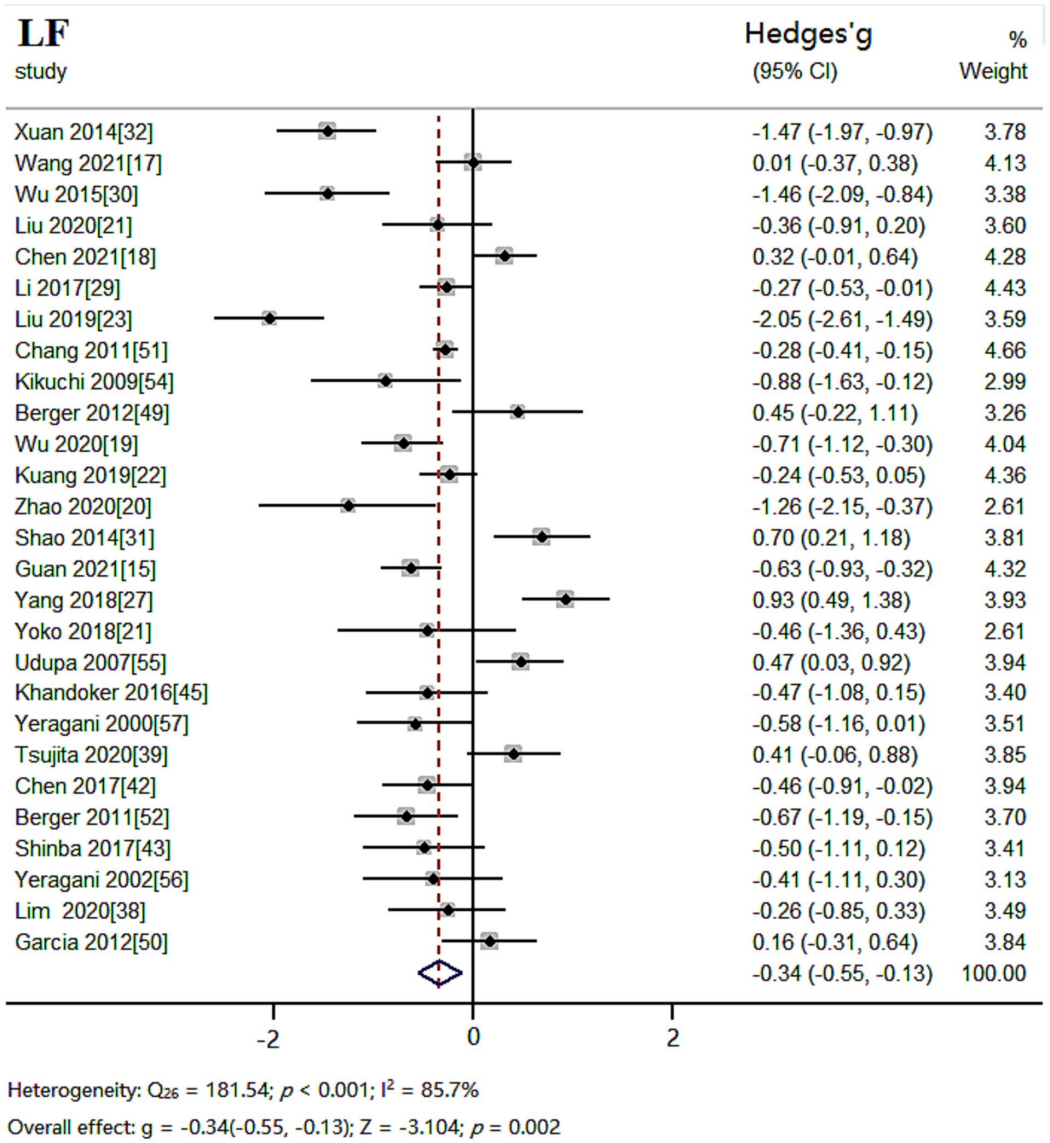
Meta-analysis forest plot of indicator LF.

The random effects model was used for meta-analysis of outcome index LF. Heterogeneity test results showed: *Q_26_* = 181.54, *p* < 0.001 and *I^2^* = 85.7%, the heterogeneity between the studies was significant. Combined effect size: *Hedges’ g* = −0.34, *95% CI* (−0.55, −0.13), *Z* = −3.104, *p* = 0.002. The effect size was small. The forest plot showed (Figure 5) that the horizontal line of the 95% CI for the indicator Hedges’ g was to the left of the null line in the depressed and healthy control groups, indicating that LF was significantly lower in the depressed group compared to the healthy control group.

#### 3.4.5 HF results

**Figure 6:**
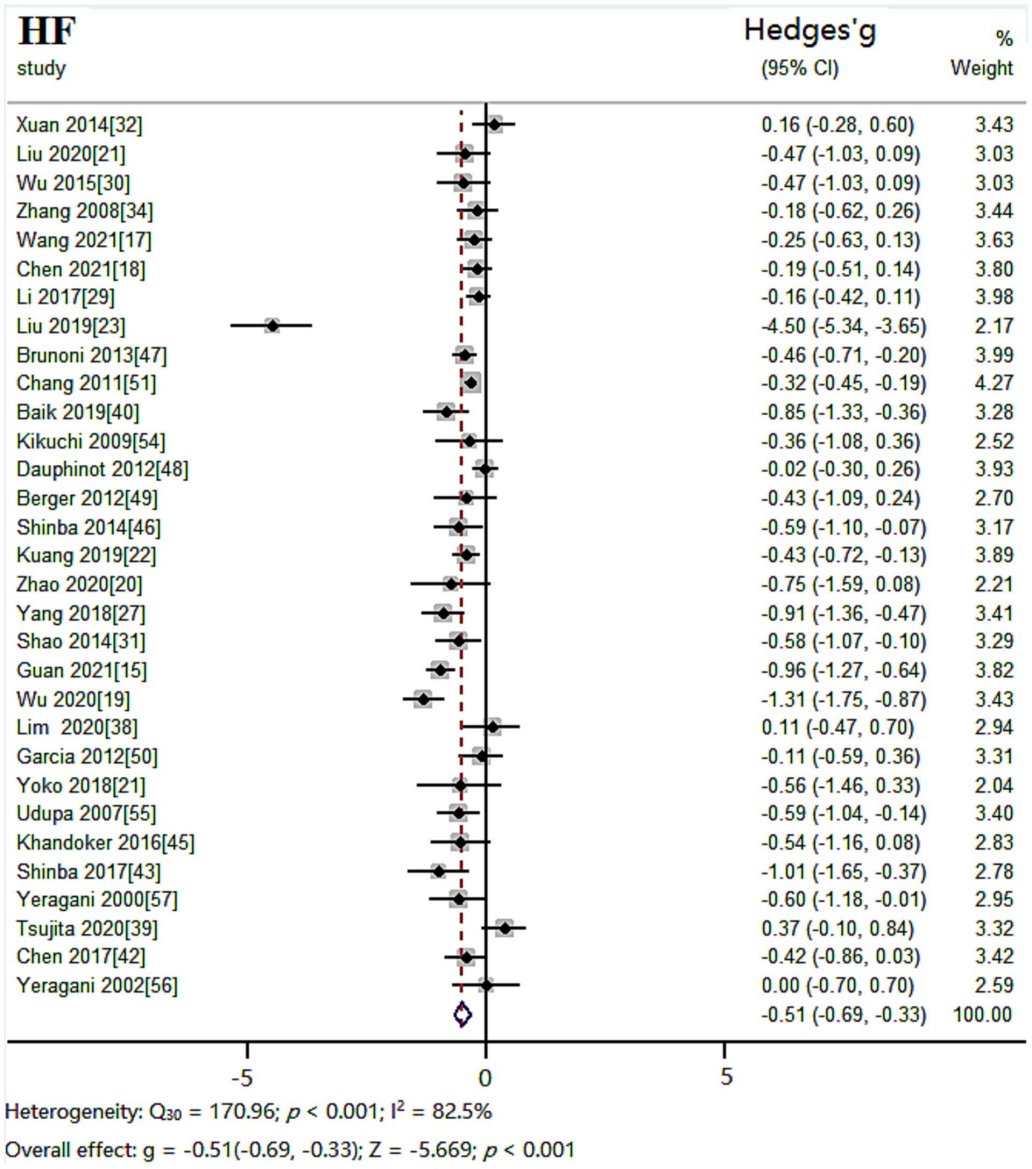
Meta-analysis forest plot of indicator HF.

The random effects model was used for meta-analysis of outcome index HF. Heterogeneity test results showed: *Q_30_* = 170.96, *p* < 0.001, *I^2^* = 82.5%, the heterogeneity between the studies was significant. Combined effect size: *Hedges’ g* = −0.51, *95% CI* (−0.69, −0.33), *Z* = −5.669, *p* < 0.001. The effect size was moderate. The forest plot showed (Figure 6) that the horizontal line of the 95% CI for the indicator Hedges’ g was to the left of the null line in the depressed and healthy control groups, indicating that HF was significantly lower in the depressed group compared to the healthy control group.

#### 3.4.6 LF/HF results

**Figure 7:**
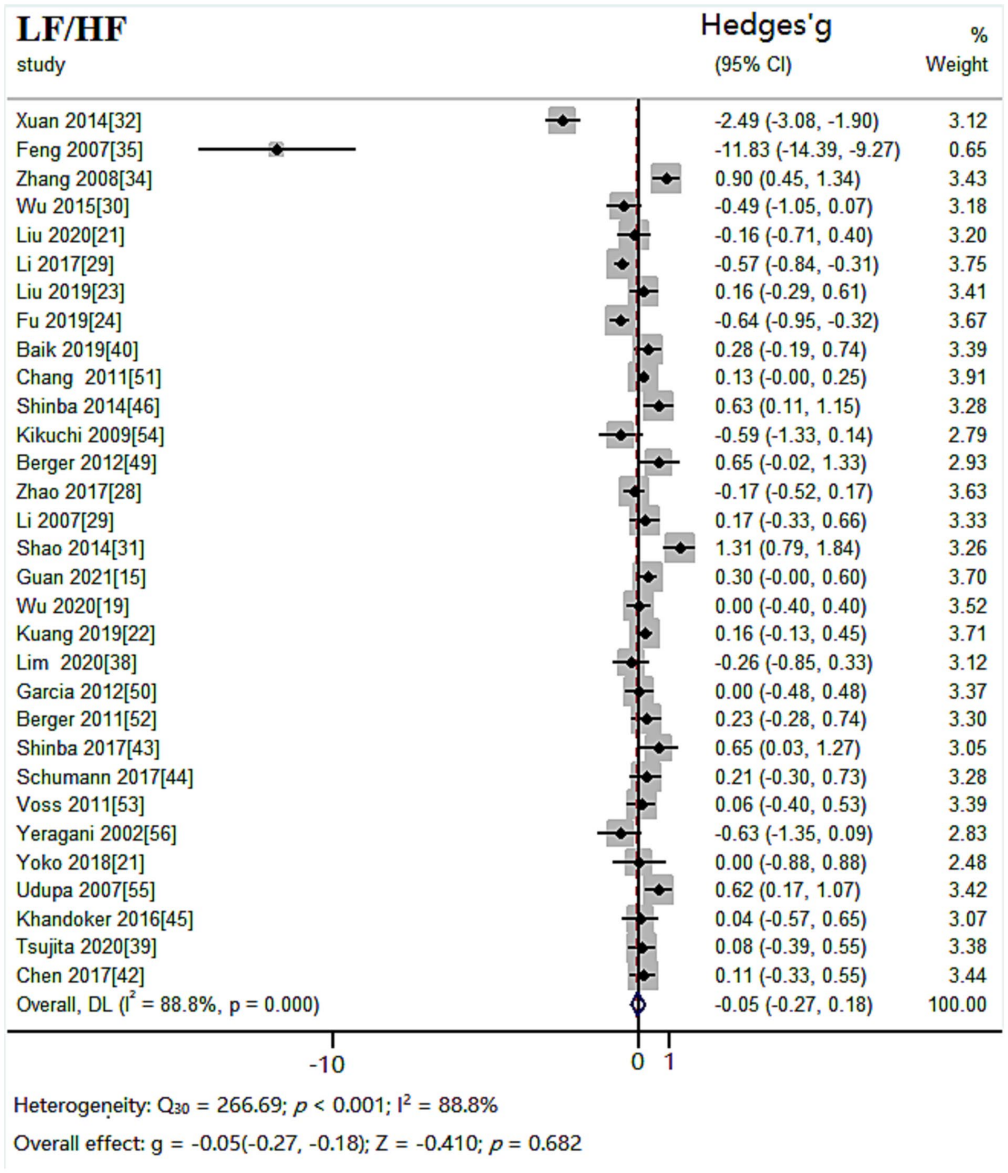
Meta-analysis forest plot of indicator LF/HF.

The random effects model was used for meta-analysis of outcome index LF/HF. Heterogeneity test results showed: *Q_30_* = 266.69, *p* < 0.001, *I^2^* = 88.8%. Combined effect size: *Hedges’ g* = −0.05, *95% CI* (−0.27,0.18), *Z* = −0.410, *p* = 0.682. The effect size was very small. The forest plot showed (Figure 7) that the 95% CI horizontal line for the indicator Hedges’ g between the depressed and healthy control groups was in the center of the null line, indicating that there was no significant difference between LF/HF in the depressed group compared to the healthy control group.

### 3.5 Results of subgroup analysis

In order to explore the source of heterogeneity, a subgroup analysis was conducted on the six indicators according to age distribution and literature sources, and the results are displayed in Tables 3, 4.

**Table 3:** Subgroup analysis of the 6 indicators by age distribution.

Ending indicators	Age	Number of studies	Heterogeneity test			Estimated value of effect		
			Q	*p*	I^2^	Hedges’ g(95%CI)	*Z*	*p*
SDNN	Youth	12	26.90	0.005^*^	59.1%	−0.50 (−0.71, −0.29)	−4.73	<0.001^**^
	Middle-aged and older adult	14	200.27	<0.001^**^	93.5%	−1.16 (−1.58, −0.73)	−5.34	<0.001^**^
RMSSD	Youth	14	27.82	0.01^*^	53.3%	−0.43 (−0.60, −0.25)	−4.75	<0.001^**^
	Middle-aged and older adult	14	123.62	<0.001^**^	89.5%	−0.61 (−0.92, −0.31)	−3.91	<0.001^**^
PNN50	Youth	7	13.10	0.042^*^	54.2%	−0.48 (−0.71, −0.25)	−4.12	<0.001^**^
	Middle-aged and older adult	6	16.85	0.005^*^	70.3%	−0.39 (−0.62, −0.15)	−3.16	0.002^*^
LF	Youth	17	82.24	<0.001^**^	80.5%	−0.2 (−0.47,0.07)	−1.46	0.144
	Middle-aged and older adult	10	95.57	<0.001^**^	90.6%	−0.57 (−0.95, −0.19)	−2.93	0.003^*^
HF	Youth	16	48.72	<0.001^**^	69.2%	−0.53 (−0.75, −0.30)	−4.60	<0.001^**^
	Middle-aged and older adult	15	112.51	<0.001^**^	87.6%	−0.50 (−0.77, −0.24)	−3.75	<0.001^**^
LF/HF	Youth	18	37.89	0.003^*^	55%	0.17 (0.01, 0.34)	2.04	0.042^*^
	Middle-aged and older adult	13	217.57	<0.001^**^	94%	−0.46 (−0.94, 0.01)	−1.92	0.054

*p < 0.05; **p < 0.001.

**Table 4:** Subgroup analysis of the 6 indicators by literature sources.

Ending indicators	Country	Number of studies	Heterogeneity test			Estimated value of effect		
			Q	*p*	I^2^	Hedges’ g(95%CI)	*Z*	*p*
SDNN	China	20	217.83	<0.001^**^	91.3%	−1.01 (−1.31,-0.70)	−6.42	<0.001^**^
	Other countries	6	7.29	0.20	31.4%	−0.26 (−0.52,-0.01)	−2.01	0.045^*^
RMSSD	China	19	138.32	<0.001^**^	87%	−0.63 (−0.88,-0.38)	−4.89	<0.001^**^
	Other countries	9	8.33	0.40	3.9%	−0.31 (−0.45,-0.16)	−4.24	<0.001^**^
PNN50	China	11	26.81	0.003^*^	62.7%	−0.48 (−0.66,-0.30)	−5.26	<0.001^**^
	Other countries	2	1.45	0.23	31.2%	−0.19 (−0.51,-0.12)	−1.20	0.229
LF	China	14	147.41	<0.001^**^	90.5%	−0.44 (−0.74,-0.14)	−2.91	0.004^*^
	Other countries	12	30.45	0.001^*^	63.9%	−0.19 (−0.47,-0.09)	−1.32	0.185
HF	China	16	138.76	<0.001^**^	89.2%	−0.65 (−0.92,-0.37)	−4.63	<0.001^**^
	Other countries	15	30.31	0.007^*^	53.8%	−0.36 (−0.55,-0.16)	−3.62	<0.001^**^
LF/HF	China	16	237.32	<0.001^**^	93.7%	−0.26 (−0.61,0.10)	−1.41	0.159
	Other countries	15	23.49	0.05	40.4%	0.17 (−0.02,0.35)	−1.76	0.078

*p < 0.05; **p < 0.001.

### 3.6 Sensitivity analysis

The included studies were eliminated one by one, and the effect size of the meta-analysis obtained after each elimination was compared to the total effect size to assess whether the results had significant changes. After using the one-by-one elimination method, it was found that after deleting Feng (35), *Q* = 76.84, *p* < 0.001, *I^2^* = 66%, *Hedges’ g* = −0.45, *95%CI* (−0.58, −0.32), although *I^2^* has decreased, there was still heterogeneity in RMSSD indicators. The results showed that after removing the article by Li (29), the heterogeneity of PNN50 index decreased significantly: *Q* = 19.45, *p* = 0.053, *I^2^* = 43.5%, and the merging result was stable: *Hedges’ g* = −0.48, *95% CI* (−0.62, −0.34), *p* < 0.001. No alterations were noticed in other indices after the one-by-one elimination method was implemented. Moreover, two effect models were also employed to assess the dependability of the meta-analysis’ outcomes. Table 5 demonstrated that the six outcome indicators did not significantly vary when the effect model was altered, indicating that the results of the meta-analysis in this investigation were relatively consistent.

**Table 5:** Comparative analysis of two effect models.

Ending indicators	Before				After			
	Model	Hedges’ g	95%CI	Q	Model	Hedges’ g	95%CI	Q
SDNN	Random	−0.87	−1.14/−0.60	270.84**	Fixed	−0.80	−0.88/−0.72	270.84**
RMSSD	Random	−0.51	−0.69/−0.33	151.63**	Fixed	−0.44	−0.51/−0.36	151.63**
PNN50	Random	−0.43	−0.59/−0.27	31.13*	Fixed	−0.41	−0.51/−0.31	31.13*
LF	Random	−0.34	−0.55/−0.13	181.54**	Fixed	−0.27	−0.34/−0.20	181.54**
HF	Random	−0.51	−0.69/−0.33	170.96**	Fixed	−0.40	−0.47/−0.33	170.96**
LF/HF	Random	−0.05	−0.27/0.18	266.69**	Fixed	0.03	−0.04/0.10	266.69**

*p < 0.05; **p < 0.001.

### 3.7 Bias analysis

**Figure 8:**
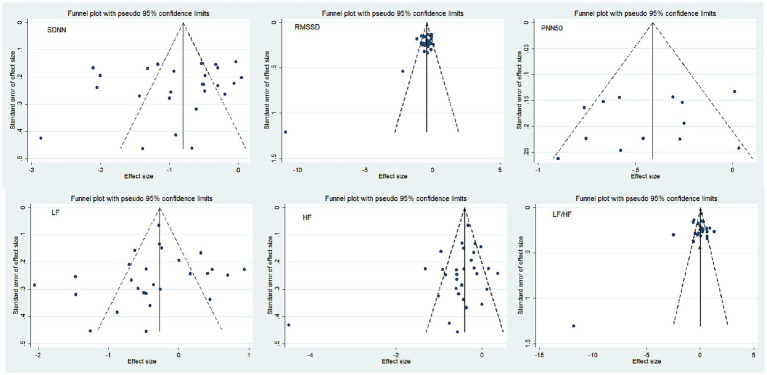
Funnel plot of publication bias.

The six outcome indicators used in this study, each of which included more than 10 papers, can be tested for publication bias by means of funnel plots (Figure 8). Since funnel plot method is a qualitative method to identify publication bias, we use the Egger’ s method. The results of Egger’ s method further showed that there was no publication bias for SDNN (*p* = 0.419), PNN50 (*p* = 0.416), LF (*p* = 0.511), HF (*p* = 0.108), and LF/HF (*p* = 0.263). While RMSSD (*p* = 0.009) had publication bias, eight research data were simulated by trim and fill procedure, Results were not significantly reversed before and after shear compensation analysis (*Hedges’ g* = −0.718, *95%CI*: −0.907, 0.528, *p* < 0.001), suggesting that the meta-analysis results were stable.

## 4 Discussion

The World Health Organization conducted a survey which showed that over 300 million people around the world are suffering from depression (58). Depression can have a significant influence on people’s quality of life and is frequently accompanied by other psychiatric conditions, particularly anxiety disorders (59), as well as somatic chronic diseases such as coronary heart disease and heart failure (60). Research revealed that patients with depression combined with coronary artery disease had increased plasma catecholamines, autonomic dysfunction, and a decline in multiple HRV markers in comparison to those with depression alone (61). A meta-analysis demonstrated that a lower HRV was associated with a higher incidence of cardiovascular disease and mortality (62). A cohort study conducted in the United States revealed that depressive symptoms are linked to the risk of heart failure events, with women being at a greater risk. This finding was further corroborated in 2014 when the American Heart Association issued a statement on depression as a risk factor for a poor prognosis in patients with acute coronary syndromes (63). Meta-analysis has indicated that an overactive hypothalamic–pituitary–adrenal axis is connected to the development of depression (64), and it can also influence the cardiovascular system of humans by modulating autonomic nerves (6). Detecting autonomic function in depressed individuals can help to protect against cardiovascular disease.

The findings of this study indicated that depressed patients displayed a significantly diminished SDNN, RMSSD, PNN50, LF, and HF in comparison to the healthy population. Additionally, Koch *et al.*’s (65) meta-analysis of HRV in patients with major depression revealed that depressed patients had notably diminished SDNN, RMSSD, LF, and HF indicators compared to the control group, which is in agreement with the current study’s results. The decrease in SDNN indexes indicates that depressed patients experience an increase in sympathetic nerve activity and a decrease in parasympathetic nerve activity, implying that they have disorders of the central nervous system’s energy supply due to external stress and heightened sensitivity to stimuli. RMSSD and PNN50 are primarily indicators of changes in vagal tone, and these can be influenced by age and gender (66). The analysis of bias revealed that there was publication bias in RMSSD, however, the results stayed nearly the same after the exclusion of the literature (35) that could have been the cause of the bias. After eliminating the study of Li (29), the heterogeneity of PNN50 index was significantly reduced, and the combined results were stable, still indicating abnormal parasympathetic activity in patients with depression. Wang et al. (67) conducted an analysis of HRV in patients with major depression and discovered that RMSSD, PNN50 and HF values were lower in the depressed group than in the control group. The imbalance in nerve activity in depressed patients, with a surge in sympathetic nerve activity and a decrease in parasympathetic nerve activity, could be the mechanism behind arrhythmia. According to Kemp et al. (68), there was no difference in LF between depressed and normal individuals. Additionally, Brown et al. (69) revealed a significant decrease in LF in older adult depressed patients when compared to healthy controls, yet no noteworthy alterations were seen in HF. For those with first-onset major depression, LF was likely to decrease and HF was likely to increase (30). The decrease in HRV predicts an imbalance in the body’s autonomic nervous system and can serve as a mediator of other psychological stress changes in depression (70), anxiety disorders (71), etc. Depression has been linked to cardiovascular disease in many cases, which is one of the most prevalent somatic comorbidities (72). According to Thayer (73), the autonomic nervous system and parasympathetic tone are connected to glucose regulation and inflammatory response, while a reduction in HRV is correlated with increased fasting glucose, nocturnal urinary cortisol, and augmented pro-inflammatory cytokines and acute phase proteins. He proposed that identifying neuroendocrine regulatory systems may aid in understanding the pathways in which psychosocial factors have an effect on health. The results of the current study did not demonstrate any significant disparity between the two groups in terms of LF/HF index. Catrambone chose subclinically depressed female patients as participants, and the LF/HF ratio was not different from that of the control group, which is in line with the findings of this study. However, other studies have reported that the LF/HF ratio (74) was higher in depressed patients than in healthy individuals (63), and the LF/HF index was significantly correlated with the Profile of Mood States (POMS) questionnaire (75).

The Q test results demonstrated a considerable degree of heterogeneity in the outcome indicators of this study, so we conducted subgroup analysis to determine the source of this heterogeneity. After conducting subgroup analysis according to age range, the LF/HF index of normal young people was found to be significantly higher than that of depressed young people, yet the heterogeneity in all indicators remained high, with no other notable changes. Ngampramuan’s study revealed that the LF/HF ratio of older adult patients with major depression was significantly higher than that of normal subjects, which differs from the results of the present study (76). Udupa (55) discovered that the LF/HF ratio was significantly higher in young people with major depression than in healthy subjects, which implies a decrease in parasympathetic activity and an increase in sympathetic activity, which is in agreement with the results of the subgroup analysis in this study. The LF/HF index has been employed for a considerable amount of time, yet other research has indicated that the LF/HF ratio may be more associated with respiratory parameters and mechanical factors, and not with alterations in cardiac autonomic nervous regulation (77). Proposals to utilize the new analytical method LF-HF for analysis (78) have been made by some academics, yet many studies presently continue to use the LF/HF index, so our research can only be evaluated based on existing studies. In the future, empirical studies and theoretical innovations should be conducted to further explore the LF/HF results, and the interpretation of these results should be done with caution. We conducted a subgroup analysis on the sources of the literature, and the results indicated that when the subjects in SDNN, RMSSD, PNN50 and LF/HF were not Chinese, the heterogeneity was not significant. This may be attributed to the differences in experimental design, environmental region, and domestic and foreign diagnostic criteria. Perhaps in the future it will be possible to directly compare the differences in heart rate variability among depressed patients in different countries or regions.

There are some shortcomings in this study. First, although we have tried our best to find the literature related to this study, there is still a possibility of missing the examination. Some grey literature was not included in this study, which may have a certain impact on the results of this study. Secondly, we only performed subgroup analysis for the age distribution range, but whether the type of depression, gender, and diagnostic criteria may have an effect on the results also deserves more detailed exploration in the future. Finally, most of the literature included in this study is based on clinical case–control data, which makes it difficult to achieve random grouping and blinded implementation, and more high-quality articles related to heart rate variability in depressed patients are expected to be published in the future.

## 5 Conclusion

In conclusion, the results of the meta-analysis of this study provide relevant evidence for the alteration of HRV in depressed patients. The utilization of the HRV test can be an effective way to assess cardiovascular health and autonomic function, which has significant implications for the prevention and surveillance of cardiovascular disease in individuals suffering from depression.

## Data availability statement

The raw data supporting the conclusions of this article will be made available by the authors, without undue reservation.

## Author contributions

QW and AC were responsible for the conception and writing of the study. QW, AC and TX were responsible for the design and revision of this study. XM and YC were responsible for the screening of the literature. All authors have read and agreed to the published version of the manuscript.
